# Perioperative management of esophagectomy in a patient who previously underwent bilateral lung transplantation

**DOI:** 10.1186/s40981-016-0041-x

**Published:** 2016-07-12

**Authors:** Hiroaki Toyama, Kazutomo Saito, Yusuke Takei, Kana Saito, Takuya Fujimine, Yutaka Ejima, Takashi Kamei, Tatsuaki Watanabe, Yoshinori Okada, Masanori Yamauchi

**Affiliations:** 1 Department of Anesthesiology, Tohoku University Hospital, 1-1 Seiryomachi, Aoba-ku, Sendai, 980-8574 Japan; 2 Division of Surgical Center and Supply, Sterilization, Tohoku University Hospital, Sendai, Japan; 3 Department of Advanced Surgical Science and Technology, Tohoku University School of Medicine, Sendai, Japan; 4 Department of Thoracic Surgery, Institute of Development, Aging and Cancer, Tohoku University School of Medicine, Sendai, Japan; 5 Anesthesiology and Perioperative Medicine, Tohoku University School of Medicine, Sendai, Japan

**Keywords:** Esophageal cancer, Esophagectomy, Bilateral lung transplantation (BLTx), Thoracoabdominal major surgery, Thoracoscopic esophagectomy, Artificial pneumothorax

## Abstract

**Background:**

General theory of anesthetic managements for nontransplant procedures in lung transplant patients was proposed. However, there are few literatures reporting the perioperative management of thoracoabdominal major surgery following lung transplantation in detail. Herein, we scrupulously report a perioperative management of esophagectomy in a patient who previously underwent bilateral lung transplantation (BLTx), focusing on protection of the transplanted lungs and the respiratory function of the patient.

**Case presentation:**

A 50-year-old woman was listed for cadaveric BLTx for severe respiratory failure due to end-stage diffuse panbronchiolitis. She underwent BLTx under veno-arterial extracorporeal membranous oxygenation support. Blood loss during the BLTx was 13,675 mL, and mild lung edema developed. She was weaned from the ventilator on the sixth postoperative day (POD) and discharged on the 65th POD. Two years after the BLTx, respiratory function improved markedly, but she was diagnosed with esophageal cancer and was scheduled for thoracoscopic esophagectomy with radical lymph node dissection, hand-assisted laparoscopic gastric mobilization, and anastomosis of the gastric conduit to the cervical esophagus via posterior mediastinum. We were concerned that impaired lymphatic drainage could cause pulmonary edema or lymphangiogenesis could cause a severe immunologic response against the lung grafts. To avoid graft injury and rejection, we addressed lung protective ventilation, reduced transfusion volume, continued immunosuppressive agents, administered volatile anesthetics, and prevented dynamic pain by epidural analgesia. These factors and the improved respiratory function may have contributed to successful management of esophagectomy. During the perioperative period, the major respiratory problems were a slight right lung edema and a persistent pulmonary air leak due to the division of thoracic adhesions, which resolved on 13th POD.

**Conclusions:**

Cancer surgeries in lung transplant recipients become more common. When such patients undergo thoracoabdominal major surgery, we should pay special attention to respiratory function, operative stress, immunosuppressive therapy, transfusion volume for the prevention of lung edema, and thoracic adhesions.

## Background

General theory of anesthetic managements for nontransplant procedures in lung transplant patients was discussed [1]. However, there are few literatures reporting the perioperative management of thoracoabdominal major surgery following lung transplantation in detail. And then, we managed an esophagectomy with radical lymph node dissection in a patient who previously underwent bilateral lung transplantation (BLTx). During the perioperative period of the esophagectomy, deterioration of expectoration and lung edema followed by respiratory dysfunction, and graft rejection were issues. Herein, we scrupulously report the perioperative management of the esophagectomy following BLTx, focusing on protection of the transplanted lungs and the respiratory function of the patient.

## Case presentation

**Fig. 1:**
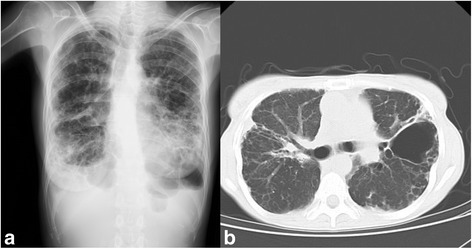
Chest radiography (**a**) and computed tomography (**b**) images of the patient before bilateral lung transplantation. **a** Significant diffuse emphysematous changes are seen on all lung fields. **b** Diffusely enhanced interstitial opacity on lung fields and an emphysematous bulla on the left lung are seen

A 50-year-old female patient was listed for cadaveric BLTx for end-stage diffuse panbronchiolitis. Her respiratory function was severely impaired and echocardiography indicated secondary pulmonary hypertension (Table 1). Chest radiography and computed tomography (CT) showed significant diffuse emphysematous changes in all lung fields (Fig. 1). She underwent BLTx under veno-arterial extracorporeal membranous oxygenation (V-A ECMO) support. Blood loss during the BLTx amounted to 13,675 mL, and mild lung edema developed. Therefore, her chest was closed on the fourth postoperative day (POD). She was weaned from the ventilator on the sixth POD and discharged ambulatory without oxygen on the 65th POD.

**Table 1:** Arterial blood gas, spirometric, and echocardiographic data of the patient before BLTx and before esophagectomy

	Before BLTx	Before MIE
Arterial blood gases on room air		
PaO_2_ (mmHg)	51	99
PaCO_2_ (mmHg)	49	44
SaO_2_ (%)	86	98.5
Spirometry		
FVC (mL)	900	2050
%FVC (%)	36.0	83.3
FEV1.0 (mL)	640	1960
%FEV1.0 (%)	71.1	95.6
Echocardiography		
LVEDd (mm)	31	41
LVEF (%)	77	75
TRPG (mmHg)	69	16
Others	TR II°	TR I°

*BLTx* bilateral lung transplantation, *MIE* minimally invasive esophagectomy, *PaO*_*2*_ arterial oxygen tension, *PaCO*_*2*_ arterial carbon dioxide tension, *SaO*_*2*_ arterial oxygen saturation, *FVC* forced vital capacity, *%FVC* percent-predicted FVC, *FEV1.0* forced expiratory volume in one second, *%FEV1.0* percent-predicted FEV1.0, *LVEDd* left ventricular end-diastolic diameter, *LVEF* left ventricular ejection fraction, *TRPG* maximum tricuspid regurgitation pressure gradient, *TR* tricuspid valve regurgitation

Two years after the BLTx, the follow-up chest CT findings suspected esophageal cancer, and the patient was diagnosed with stage II esophageal cancer via esophagogastroduodenoscopy. Three months later, she was scheduled for thoracoscopic esophagectomy with radical lymph node dissection, hand-assisted laparoscopic gastric mobilization, and anastomosis of the gastric conduit to the cervical esophagus via posterior mediastinum.

**Fig. 2:**
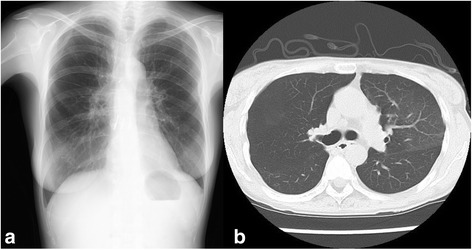
Chest radiography (**a**) and computed tomography (**b**) images of the patient before minimally invasive esophagectomy. **a** Normalized lung fields, and sternal wires and marking clips on mediastinal field are seen. **b** Normalized lung fields and marking clips on the esophagus are seen

She was 147 cm tall and weighed 35 kg. Before esophagectomy, her respiratory function was normal and her echocardiography indicated disappearance of secondary pulmonary hypertension and subsequent increased left ventricular preload (Table 1). Chest radiography and CT showed normalized lung fields (Fig. 2), but postoperative intrathoracic adhesions were suspected. Her preoperative blood examination detected no abnormal values except for a slightly elevated plasma creatinine concentration of 1.02 mg/dL and decreased hemoglobin concentration of 9.8 g/dL. And white blood cell (WBC) counts was 4100 /μL, and serum C-reactive protein (CRP) level was 0.1 mg/dL. She was treated with immunosuppressive agents including 1.6 mg/day of tacrolimus (blood concentration was 6.0 ng/mL), 500 mg/day of mycophenolate mofetil, and 5 mg/day of prednisolone, with 0.625 mg/day of bisoprolol for the suppression of right heart function. On the morning of surgery, she was administered 500 mg of methylprednisolone intravenously for the prevention of rejection and relative adrenal insufficiency. In the operating room, a thoracic epidural catheter was placed at the level of the T9–T10 intervertebral space. Before anesthesia induction, arterial catheter was inserted and then phenylephrine infusion (0.25 μg/kg/min) was initiated to prevent hypotension induced by anesthetic agents. After oxygenation, general anesthesia was induced by continuous infusion of remifentanil (0.5 μg/kg/min), intravenous bolus administrations of propofol (20 mg), and rocuronium (30 mg). She was orotracheally intubated with a 35 French double-lumen endobronchial tube. Due to the significant incidence of complications on bronchial anastomosis, endobronchial tube was positioned with fiberoptic bronchoscopic guidance. After intubation, central venous catheter was inserted via left basilic vein and the monitoring of central venous pressure (CVP) was started. During induction and intubation, hemodynamic and respiratory status remained stable. She received pressure-controlled ventilation with a fraction of inspired oxygen (F_I_O_2_) of 0.3–0.45, positive end-expiratory pressure (PEEP) of 5 cmH_2_O, peak inspiratory pressure (PIP) of less than or equal to 18 cmH_2_O, and respiratory rate (RR) of 10–18/min. During two-lung ventilation, the dynamic compliance (Cdyn) and the arterial oxygen tension to F_I_O_2_ ratio (P/F) of her lungs were approximately 30 mL/cmH_2_O and more than 500, respectively, and the CVP was 4–8 mmHg. Anesthesia was maintained by desflurane (3.5 %) and remifentanil (0.5–0.7 μg/kg/min), with continuous epidural analgesia using 0.25 % levobupivacaine (4 mL/h). Phenylephrine (0.1–0.4 μg/kg/min) and noradrenaline (0.03–0.04 μg/kg/min) infusion were used to prevent hypotension and overhydration, and sivelestat infusion (0.4 mg/kg/h) and carperitide infusion (10 ng/kg/min) were used to reduce lung injury and extravascular lung water. She was placed in the left lateral position and thoracoscopic esophagectomy with radical lymph node dissection was started. One-lung ventilation and an artificial pneumothorax, insufflating with carbon dioxide gas (CO_2_) to promote lung collapse, were started, and then the CVP increased to 12–18 mmHg. During the thoracic procedure, the Cdyn and P/F of her lung were 15–20 mL/cmH_2_O and 273–421, respectively, with PaCO_2_ and serum lactate level elevated up to 63 mmHg and 2.7 mg/dL, respectively. Artificial pneumothorax was prolonged (396 min), because of extensive intrathoracic adhesions, and a pulmonary air leak persisted after the thoracic procedure. After that, two-lung ventilation was resumed, and she was placed in the lithotomy position for abdominal and cervical procedure. Her CVP decreased to 4–7 mmHg. Hand-assisted laparoscopic gastric mobilization was started, which increased the CVP to 10–12 mmHg, transiently. Then, the gastric conduit was anastomosed with the cervical esophagus via the posterior mediastinum and a jejunostomy tube was placed for enteral feeding. One hour before the completion of the surgery, desflurane was discontinued, propofol (4 mg/kg/h) was started, and remifentanil was reduced to 0.3 μg/kg/min. After the completion of the surgery, bronchoscopy and arterial blood gases confirmed the preservation of bronchial blood flow and pulmonary function, and maintenance anesthetics and phenylephrine were discontinued. She recovered from the anesthesia and her tracheal tube was removed. Duration of the surgery and anesthesia were 669 min and 760 min, respectively. Blood loss and urine volume during anesthesia were 217 mL and 775 mL, respectively. We infused with 550 mL of 6 % hydroxyethyl starch 130/0.4 solution, 500 mL of amino acid solution, 650 mL of saline, and 120 mL of acetated Ringer’s solution during anesthesia. At the completion of the surgery, her serum creatinine and lacate values were 1.4 mg/dL and 2.1 mg/dL. Subsequently, she was transferred to the intensive care unit with continuous epidural analgesia of 4 mL/h of levobupivacaine 0.25 % + fentanyl 2.5 μg/mL and patient controlled epidural analgesia of bolus 3 mL (30-min lock out).

**Fig. 3:**
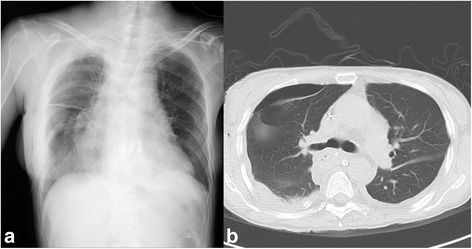
Chest radiography (**a**) and computed tomography (**b**) images of the patient on the 3rd postoperative day. **a** Chest drainage tube, permeability decay, and expansion failure on the right lung fields, subcutaneous emphysema on the right chest wall, drainage tube and gastric conduit on the mediastinal field, and peripheral venous nutrition tube on the left arm are seen. **b** Chest drainage tube, moderate pleural effusion, and expansion failure on the right lung, and small pleural effusion on the left lung are seen

**Fig. 4:**
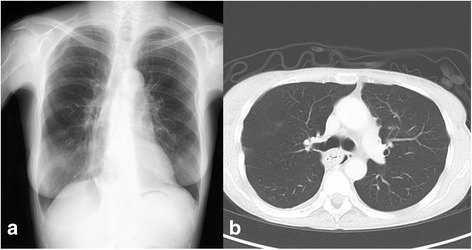
Chest radiography (**a**) and computed tomography (**b**) images before discharge. **a** Reexpanded right lung and gastric conduit on the mediastinal field are seen. **b** Reexpanded right lung, disappearance of pleural effusion, and gastric conduit on the right posterior mediastinum are seen

On the first POD, WBC count and CRP level were elevated to the level indicating moderate-to-intense inflammatory reaction, and the CVP remained 4–6 mmHg. Table 2 shows the changes of her WBC counts, serum CRP, creatinine and lactate levels, and water balance during perioperative period. Intravenous methylprednisolone 125 mg, cyclosporin infusion (2 mg/h, 250–300 ng/mL of target blood concentration), and enteral mycophenolate mofetil (500 mg/day) were administered. And noradrenaline was discontinued. On the second POD, her inflammatory reaction was persisted. Cyclosporin infusion was discontinued and enteral tacrolimus (1.6 mg/day, 8–10 ng/mL of target blood concentration) was initiated. On the third POD, her body weight increased to a maximum value of 37.7 kg due to daily positive water balance, and chest radiography and CT showed slight pulmonary edema and pneumothorax in the right lung field (Fig. 3). However, her inflammatory reaction attenuated and CVP increased to 14–18 mmHg. Sivelestat infusion was discontinued and blood concentration of tacrolimus was 7.4 ng/mL. On the fourth POD, her body weight decreased to 36.7 kg and CVP decreased to 10–12 mmHg due to a beginning of intravascular refilling and diuretic phase. On the ninth POD, moderate inflammatory reaction was continued and her blood concentration of tacrolimus was maintained within 9.0 to 11.7 ng/mL. And she started oral dietary intake. On the 11th POD, carperitide was discontinued. On the 13th POD, inflammatory reaction diminished. And her chest drainage tube was removed and intravenous methylprednisolone was tapered slowly and then discontinued. She was discharged ambulatory without oxygen on the 29th POD. Before discharge, chest radiography and CT showed the disappearance of pulmonary edema and pneumothorax (Fig. 4).

**Table 2:** Postoperative white blood cell counts, serum C-reactive protein, creatinine and lactate levels, and water balance during perioperative period

POD	−1	0	1	2	3	5	6	9	13	29
WBC (/μL)	4100	29,600	16,000	19,000	12,800	11,000	10,800	11,000	7000	7300
CRP (mg/dl)	0.1	–	5.4	4.0	1.7	1.3	1.1	0.6	0.7	0.1
Creatinine (mg/dl)	1.02	1.4	1.1	1.0	1.0	0.86	1.0	1.18	1.16	1.03
Lactate (mg/dl)	1.3	2.1	1.57	1.06	1.0	–	–	–	–	–
Infusion volume (mL)	–	2692	2982	2735	1608	2340	2340	2040	1290	–
Water output (mL) (Urine (mL))	– –	1511 (1090)	1339 (1013)	1645 (1403)	2680 (2280)	1978 (1548)	1758 (1368)	1429 (1369)	1867 (1867)	– –
Body weight (kg)	35.0	–	35.1	36.6	37.7	36.7	36.5	35.9	35.8	–

*POD* postoperative day after esophagectomy, *WBC* white blood cell counts, *CRP* serum C-reactive protein level

### Discussion

Solid organ transplant recipients have elevated cancer risk due to immunosuppression [2, 3]. As a result, cancer surgeries in lung transplant recipients may increase along with the increase in lung transplants. During these surgeries, protection of the lung graft is the top priority, because the 5-year survival for the recipients of cadaveric donor lung transplants is approximately 50 % [4], which is significantly lower than that of other organ transplants but similar to that of patients with stage II esophageal cancer.

During lung transplantation, anastomosis of donor lymphatic vessels to those of the recipient is not performed. Therefore, pulmonary edema and acute rejection readily occur for several days after transplantation. The graft lymphatics establish new connections with regional lymph nodes no earlier than at least 7 days [5, 6]. Subsequently, lymphatic drainage would remove excess lung fluid and damaging substances, which would improve graft outcome [7]. However, the extent of lymphangiogenesis after transplantation might correlate with the magnitude of donor antigen presentation and may influence the severity of the immunologic response against the donor organ [8, 9]. Therefore, we took great care to avoid enhancement of her immunological response, which could induce graft injury and rejection. The measures were as follows: (1) lung protective ventilation with F_I_O_2_ ≤ 0.6, PEEP = 5 cmH_2_O, PIP ≤ 18 cmH_2_O; (2) the continuation of immunosuppressive agents and loading of methylprednisolone during the perioperative period; (3) neutrophil elastase inhibitor (sivelestat) infusion; (4) use of volatile anesthetics (desflurane) preventing inflammation for the maintenance of anesthesia [10]; and (5) adequate control of pain using epidural analgesia. Neuraxial anesthesia is considered to facilitate coughing, expectoration, and active mobilization, and prevent atelectasis and respiratory infections, although neuraxial anesthesia reduce intercostal muscle strength. Furthermore, thoracic epidural analgesia blocks sympathetic nervous system, which inhibits inflammatory reaction supplementarily. Although these measures were used, inflammatory reaction raised maximum level (WBC 19,000/μL and CRP 5.4 mg/dL), indicating moderate-to-intense inflammatory reaction, on the first to second POD and continued until the 13th POD. Therefore, slight right lung edema was observed but graft injury and rejection were not observed.

Transplanted lungs are highly susceptible to fluid overload over a long period. Lymphatic interruption increases the risk of extravascular lung water accumulation [11] and it may be responsible for pulmonary edema development following even minimal fluid overload [12]. In this case, lymph node dissection, which removed newly established graft lymphatics, division of thoracic adhesions, and the placement of gastric conduit, which passed through the right posterior mediastinum, were performed. These procedures had the potentials to induce interstitial lung edema and subsequent pulmonary dysfunction, because of encouraging inflammation of lung tissue, hemorrhage, prolonged surgery, and increased transfusion. To reduce the transfusions and maintain low central venous pressure, which inhibit the increase in interstitial lung water [13], we used phenylephrine and noradrenaline for maintenance of adequate blood pressure, and carperitide for excretion of excess lung fluid during anesthesia. These treatments may have contributed to prevent further deterioration of the right lung edema and the progression to pulmonary dysfunction or rejection. In this case, we monitored arterial blood pressure and CVP for the assessment of hemodynamics, but CVP, fluctuating with changes of intrathoracic pressure, was inaccurate index of cardiac preload. Therefore, transpulmonary thermodilution technique might be better to assess the hemodynamics of patients like this case. But we did not use transpulmonary thermodilution technique in this case, because the cardiac function of the patient was normal and lithotomy position during abdominal manipulation could cause femoral arterial catheter kinking, although the measurement of extravascular lung water and pulmonary vascular permeability index would be useful in the postoperative managements of the patient. Furthermore, the reduction in blood flow to her bronchi during esophagectomy was of concern because the bronchial arteries had been eliminated from her lung. However, bronchoscopy during perioperative surgery showed the preservation of blood flow to her bronchi. Whereas, intravascular refilling and diuretic phase occurred in comparatively later POD (during the third POD and fourth POD). This might be because the dissection of the lung graft lymph node impaired the lymphatic drainage and removal of excess lung fluid.

The respiratory function of this patient improved significantly after BLTx. In two-lung ventilation during esophagectomy, her Cdyn, P/F, and ratio of dead space to tidal volume were within normal limit (30 mL/H_2_O, 500, and 0.15, respectively). Therefore, ventilatory insufficiency did not occur during artificial pneumothorax and pneumoperitoneum, although her PaCO_2_ was elevated transiently. However, in patients with single lung transplantation and/or poor lung graft function, ventilatory insufficiency might occur, which promotes ventilator-induced lung injury and an inflammatory reaction, followed by pulmonary dysfunction and graft rejection. During the perioperative period of esophagectomy, the major respiratory problems of this case were a slight right lung edema and a persistent pulmonary air leak due to the division of the thoracic adhesions, which needed 13 days to recover.

## Conclusions

We report the perioperative management of a patient receiving esophagectomy who previously underwent BLTx. Cancer surgeries in lung transplant recipients increase. When such patients undergo thoracoabdominal major surgery as this case, we should pay special attention to respiratory function, operative stress, immunosuppressive therapy, volume of transfusion for prevention of lung edema, and thoracic adhesions.

## Consent

Written informed consent was obtained from the patient for publication of this Case report and any accompanying images. A copy of the written consent is available for review by the Editor-in-Chief of this journal.

## Abbreviations

BLTx, bilateral lung transplantation; Cdyn, dynamic compliance; CO_2_, carbon dioxide gas; CT, computed tomography; CVP, central venous pressure; F_I_O_2_, fraction of inspired oxygen; P/F, arterial oxygen tension to F_I_O_2_ ratio; PEEP, positive end-expiratory pressure; PIP, peak inspiratory pressure; POD, postoperative day; RR, respiratory rate; V-A ECMO, veno-arterial extracorporeal membranous oxygenation
